# Mechanistic insights into the lncRNA-Notch signaling axis in tumors

**DOI:** 10.3389/fcell.2026.1903203

**Published:** 2026-07-22

**Authors:** Qingmiao Shi, Na Lou, Huiwu Xing, Leiya Fu

**Affiliations:** 1 Department of Infectious Diseases, The First Affiliated Hospital, College of Clinical Medicine, Henan University of Science and Technology, Luoyang, China; 2 Henan Medical Key Laboratory of Gastrointestinal Microecology and Hepatology, Luoyang, China; 3 Department of Infectious Diseases, The First Affiliated Hospital of Zhengzhou University, Zhengzhou, China; 4 Department of Pediatric Surgery, The First Affiliated Hospital of Zhengzhou University, Zhengzhou, China

**Keywords:** competitive endogenous RNA, long non-coding RNA, mechanism, Notch signaling pathway, tumor

## Abstract

Cancer remains a major global health burden, with its incidence and mortality rates persistently high despite advances in treatment. Despite therapeutic innovations, malignant tumors continue to pose a formidable challenge to global health. Against this backdrop, the crosstalk between long non-coding RNAs (lncRNAs) and the Notch signaling pathway has emerged as a pivotal driver of tumorigenesis and progression. However, the complex regulatory network and a comprehensive mechanistic framework of this axis await systematic elucidation. This review systematically consolidates recent advances in understanding how lncRNAs precisely modulate Notch pathway activity through diverse mechanisms, including acting as competing endogenous RNAs, direct protein binding, epigenetic regulation, and exosome-mediated intercellular communication. The discussion encompasses various malignancies, spanning the digestive, respiratory, urogenital, nervous, and hematologic systems. The lncRNA-Notch regulatory axis is identified as a ubiquitous and functionally central oncogenic network. It orchestrates critical malignant phenotypes—such as such as stemness maintenance, epithelial-mesenchymal transition, metabolic shifts, drug resistance, and immune evasion—through intricate bidirectional crosstalk. Functional studies confirm that targeting key nodes of this axis can effectively reverse drug resistance and suppress tumor growth. Although challenges remain in its clinical translation, future research integrating single-cell multi-omics, nanotechnology, and other innovative strategies will undoubtedly open new avenues for precision diagnosis and cancer therapy.

## 1 Introduction

Cancer remains a major global public health challenge with persistently high incidence and mortality. Epidemiological data indicate that age is a key risk factor for cancer development, and global population aging is expected to further increase the cancer burden (Chen et al., 2023; Siegel et al., 2022). In the United States alone, an estimated two million new cancer cases and over 600,000 deaths were reported in 2022. Global statistics from 2018 revealed approximately 18.1 million new cases and 9.6 million deaths worldwide, with mortality rates projected to continue rising. Despite significant advances in medical science and technology, including early detection, targeted therapies, and surgical interventions that have brought cancer treatment under some control, it remains one of the most difficult diseases to treat (Cheng Z. et al., 2021; Hashemi et al., 2022).

Accumulating evidence demonstrates that tumorigenesis is tightly governed by conserved cellular signaling pathways. Among them, the Notch pathway stands out for its well-documented roles in oncogenesis, tumor progression, and therapeutic resistance—making it a compelling candidate for targeted intervention. The Notch signaling pathway is an evolutionarily conserved cell-cell communication system, comprising four Notch receptors (Notch1-4), five ligands (JAG1, JAG2, DLL1, DLL3, DLL4). Its downstream transcriptional regulatory complexes include mastermind-like (MAML) and CBF1/Suppressor of Hairless/Lag-1—also designated as recombination signal binding protein for immunoglobulin kappa J region (RBP-J) or collectively termed CSL (Ferreira and Aster, 2022; Shi et al., 2024). Its canonical activation mechanism relies on direct cell-to-cell contact: the binding of a ligand on the surface of a signal-sending cell to a Notch receptor on an adjacent signal-receiving cell triggers sequential proteolytic cleavages of the receptor (mediated by ADAM metalloprotease and γ-secretase complex) (Siebel and Lendahl, 2017). This process releases the Notch intracellular domain, which translocates to the nucleus (D’Assoro et al., 2022), forms a transcriptional activation complex with proteins such as CSL, and initiates the transcription of target genes like HES and HEY, thereby regulating critical cellular processes including proliferation, differentiation, and apoptosis. In cancer biology, the Notch pathway exhibits a context-dependent dual role, capable of either promoting or suppressing tumor progression (Wang et al., 2024; Zhou et al., 2022). Its function is determined by factors such as cell type, tissue microenvironment, interacting signaling pathways, and specific receptor-ligand pairs. For example, in colorectal cancer (CRC), Notch2 directly promotes malignant proliferation of tumor cells by transcriptionally activating TCF7L2 and its downstream target genes MYC and JUN (Xiang et al., 2025). In contrast, in skin cancer, Notch1 exerts a crucial tumor-suppressive role by transcriptionally repressing the beta-catenin signaling pathway and blocking the sustained expression of the Sonic hedgehog effector Gli2 (Nicolas et al., 2003). In response to aberrant Notch signaling activation, various therapeutic strategies have been developed, including gamma-secretase inhibitors, blocking antibodies targeting specific receptors or ligands, and ADAM protease inhibitors (Li et al., 2023; You et al., 2023).

Long non-coding RNAs (lncRNAs) have emerged as pivotal players in cancer biology in recent years. Extensive studies demonstrate that lncRNAs, through mechanisms including chromatin modification, transcriptional and post-transcriptional regulation, play critical oncogenic or tumor-suppressive roles in virtually all cancer hallmarks, with their dysregulation widely implicated in tumor initiation, progression, metastasis, and therapy resistance (Luo et al., 2026; Shen et al., 2026; Su et al., 2018; Yang et al., 2026). Importantly, lncRNAs do not function in isolation but exert their influence by precisely modulating multiple core oncogenic signaling pathways. Research indicates that lncRNAs integrate and coordinate key pathways such as Wnt/beta-catenin, TGF-β, and Notch to collectively drive cancer progression (Li et al., 2023; Rodrigues-Junior et al., 2026; Zhang N. et al., 2026). Indeed, the functional coupling between lncRNAs and the Notch signaling pathway represents an increasingly recognized regulatory axis. For instance, studies in colorectal cancer suggest that lncRNAs modulate Notch pathway activity, thereby influencing the epithelial-mesenchymal transition (EMT) program and metastatic potential (Emam et al., 2022). This interaction is particularly crucial in maintaining the plastic state of tumor cells. Although existing research has revealed specific cases of LncRNA-mediated regulation of the Notch pathway across various cancers (El-Ashmawy et al., 2024), recent work focusing specifically on triple-negative breast cancer (TNBC) has preliminarily demonstrated the importance of this axis in governing cancer stemness maintenance, EMT, metabolic reprogramming, and chemoresistance within this aggressive subtype (Shi et al., 2025; Zhang et al., 2021c), However, current knowledge remains relatively fragmented and lacks systematic mechanistic integration.

## 2 Literature search strategy

To ensure comprehensive literature coverage and minimize potential selection bias while maintaining the narrative scope of this study, a structured literature search was executed across the PubMed and Web of Science databases from database inception up to June 2026. The search strategy utilized targeted combinations of keywords and text words, including “long non-coding RNA,” “lncRNA,” “Notch signaling,” and “tumors,” specifically focusing on capturing studies that elucidate the core molecular mechanisms, regulatory networks, and translational applications of this axis. To align with the methodological transparency expected by high-impact journals, the identification and selection of relevant literature followed a streamlined framework adapted from recent benchmarking templates for reporting search methods in mechanistic reviews (Verma et al., 2025). Literature curation was governed by explicit criteria: peer-reviewed original research articles and authoritative reviews focusing on lncRNA-Notch crosstalk in human malignancies or preclinical models were prioritized, whereas conference abstracts, letters, and incomplete datasets were excluded. Preprints were scrutinized for data integrity and only considered when peer-reviewed versions were entirely unavailable but the experimental methodology was exceptionally robust. Given the descriptive and conceptual nature of our narrative review, formal PRISMA guidelines and systematic quality assessment tools were not applied; nevertheless, this explicitly outlined search strategy provides a clear, reproducible, and rigorous synthesis that accurately reflects the current state of precision oncology.

## 3 Overview of LncRNA

LncRNAs are broadly defined as a diverse class of RNA transcripts longer than 200 nucleotides that lack protein-coding potential (Watmuff et al., 2025). The human genome transcribes a vast number of these molecules, which exhibit characteristics similar to mRNAs, such as RNA polymerase II-dependent transcription, 5′ capping, splicing, and polyadenylation, yet they generally display lower expression levels and higher tissue specificity (Derrien et al., 2012). LncRNAs are categorized based on their genomic position relative to protein-coding genes, including intergenic (lincRNAs), antisense, intronic, and enhancer-associated transcripts. Their functional mechanisms are highly versatile, acting as signals, decoys, guides, or scaffolds to regulate chromatin architecture, transcription, and post-transcriptional processes. A prime example is XIST, a well-studied lncRNA essential for X-chromosome inactivation in female mammals, which orchestrates gene silencing across an entire chromosome by recruiting repressive complexes (Almeida et al., 2026; Navarro-Cobos and Brown, 2025; Zhang et al., 2025). The intricate roles of lncRNAs extend to the formation of nuclear bodies, such as paraspeckles, where they serve as architectural scaffolds to sequester proteins and RNAs, thereby influencing gene expression programs in response to cellular stimuli. Furthermore, dysregulation of specific lncRNAs is intricately linked to human diseases, including cancer and neurological disorders, highlighting their significant potential as diagnostic biomarkers and therapeutic targets (Chen and Kim, 2024; Fei et al., 2024; Wang et al., 2023; Wang H. et al., 2026). Ongoing research continues to unravel the complexity of lncRNA biology, revealing their critical functions in development and cellular homeostasis.

LncRNAs serve as pivotal regulators in tumorigenesis and cancer progression, exerting their influence through epigenetic modifications, signaling pathway interventions, and cellular fate determination. In colorectal cancer, lncRNAs promote metastasis by regulating epithelial-mesenchymal transition EMT-related transcription factors and key pathways such as Notch and Wnt; their high stability in body fluids also positions them as promising non-invasive diagnostic biomarkers (Hamidi et al., 2022). In breast cancer, HOTAIR drives metastasis by repressing miR-7 and enhancing SETDB1 expression, while downregulated MEG3 activates Notch/PI3K pathways, fostering tumor proliferation and angiogenesis (Zhang et al., 2014). Notably, lncRNA functions exhibit tissue-specificity: MIR22HG exerts tumor-suppressive effects in colorectal cancer and hepatocellular carcinoma by adsorbing oncogenic microRNAs and inhibiting pro-oncogenic pathways such as the Wnt/beta-catenin pathway, while in glioblastoma, it acts as the host gene of miR-22 to process and generate oncogenic miR-22. Accordingly, it exhibits a dual regulatory characteristic characterized by upregulation in the former cancer types and downregulation in the latter, along with opposite biological effects (Han et al., 2020; Wu Y. et al., 2019; Xu et al., 2020a; Zhang L. et al., 2020). Conversely, tumor-suppressive lncRNAs like LINC00261 inhibit cancer progression by suppressing cell proliferation, motility, and chemoresistance, correlating with favorable patient prognosis (He et al., 2025; Wang X. et al., 2020; Zhang et al., 2021e). This dichotomous nature underscores the complexity of lncRNA-mediated regulation.

Driven by their functional diversity and complexity, lncRNAs act as pivotal regulators of the Notch signaling pathway through various molecular mechanisms. As previously discussed, Notch signaling is highly conserved in cell fate determination, and the precise orchestration of its activity relies heavily on the involvement of lncRNAs. The competing endogenous RNA (ceRNA) crosstalk represents a highly prevalent intracellular regulatory mode, wherein lncRNAs act as molecular sponges to sequester target miRNAs, thereby alleviating their repressive effects on core Notch components (Zhang L. et al., 2026). As an illustration, oncogenic lncRNAs like PVT1 sponge miR-146a to upregulate Notch1 and promote tumor progression (Liu et al., 2015; Sayed et al., 2025). Beyond RNA-RNA interactions, lncRNAs can directly bind to Notch signaling proteins to alter their functional states. For instance, BREA2 interferes with the ubiquitination and degradation of the Notch intracellular domain (NICD), maintaining its stability and sustaining downstream transcriptional activity (Zhang et al., 2023). Furthermore, lncRNAs exert epigenetic modulation by guiding chromatin-remodeling complexes to the promoters of Notch ligands or receptors (Zeng et al., 2021). At a macroscopic level, exosomal lncRNAs extend this regulation beyond individual cells, mediating critical intercellular crosstalk to reprogram the tumor microenvironment (Marima et al., 2024). Notably, tumor-derived small extracellular vesicles (sEVs/exosomes) can selectively package lncRNAs along with epigenetic modifiers or metabolites, enabling horizontal epigenetic rewiring of recipient cells without altering their genome sequence—a mechanism recently framed as “exosome-mediated epigenetic inheritance” in cancer (Verma et al., 2026). To comprehensively understand how lncRNAs dissect and orchestrate the lncRNA/Notch axis in cancer, the subsequent sections will systematically elaborate based on their distinct mechanistic modes.

## 4 Involvement of lncRNAs and Notch pathway in tumors

**FIGURE 1:**
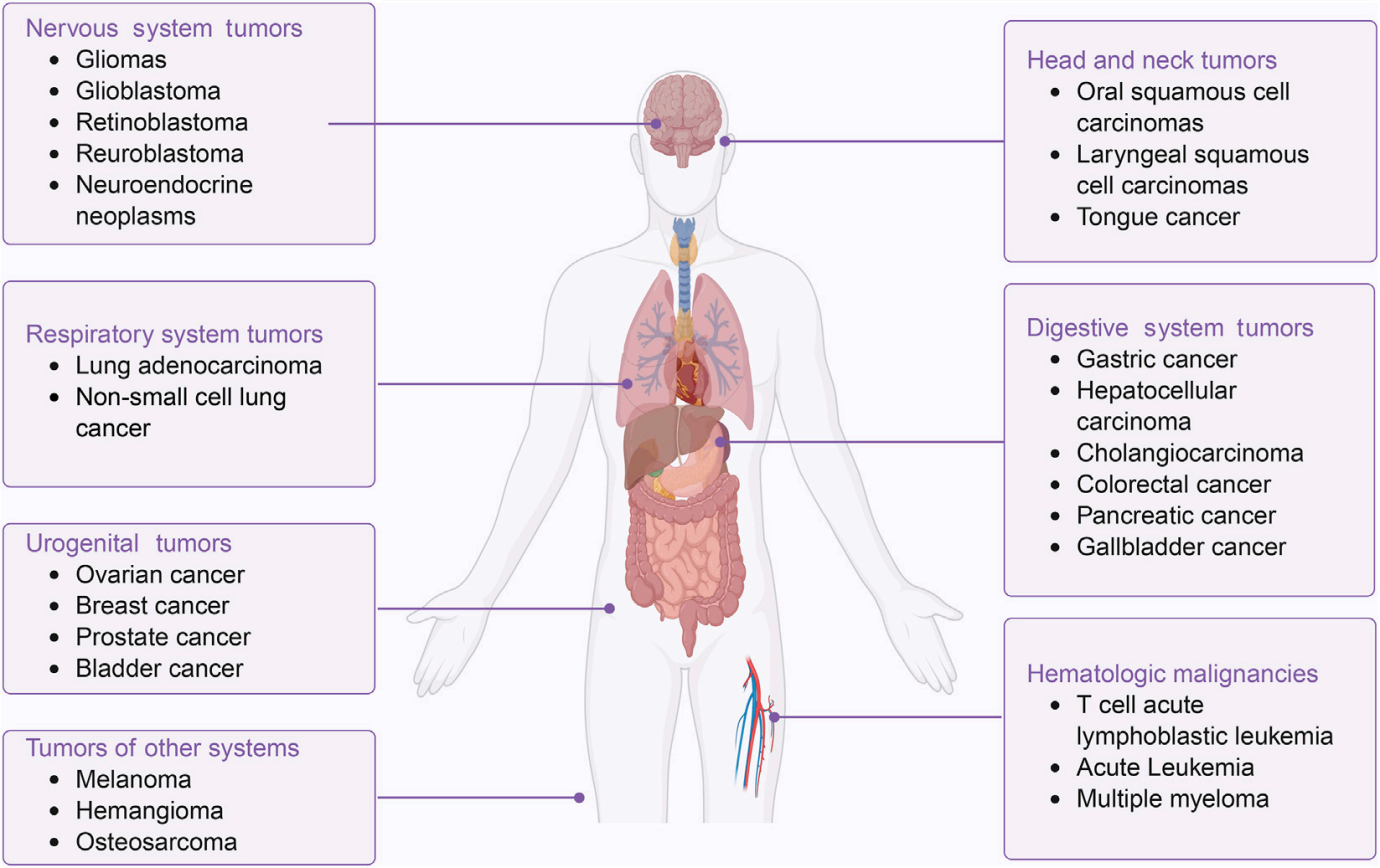
Involvement of Notch signaling pathway-associated lncRNAs in the regulation of diverse cancers. The functional crosstalk between lncRNAs and the Notch signaling pathway plays a critical role in the oncogenesis and progression of various human cancers. These encompass nervous system tumors, respiratory system tumors, urogenital tumors, head and neck tumors, digestive system tumors, hematological malignancies, as well as tumors in other systems (Figure created using BioRender.com).

**FIGURE 2:**
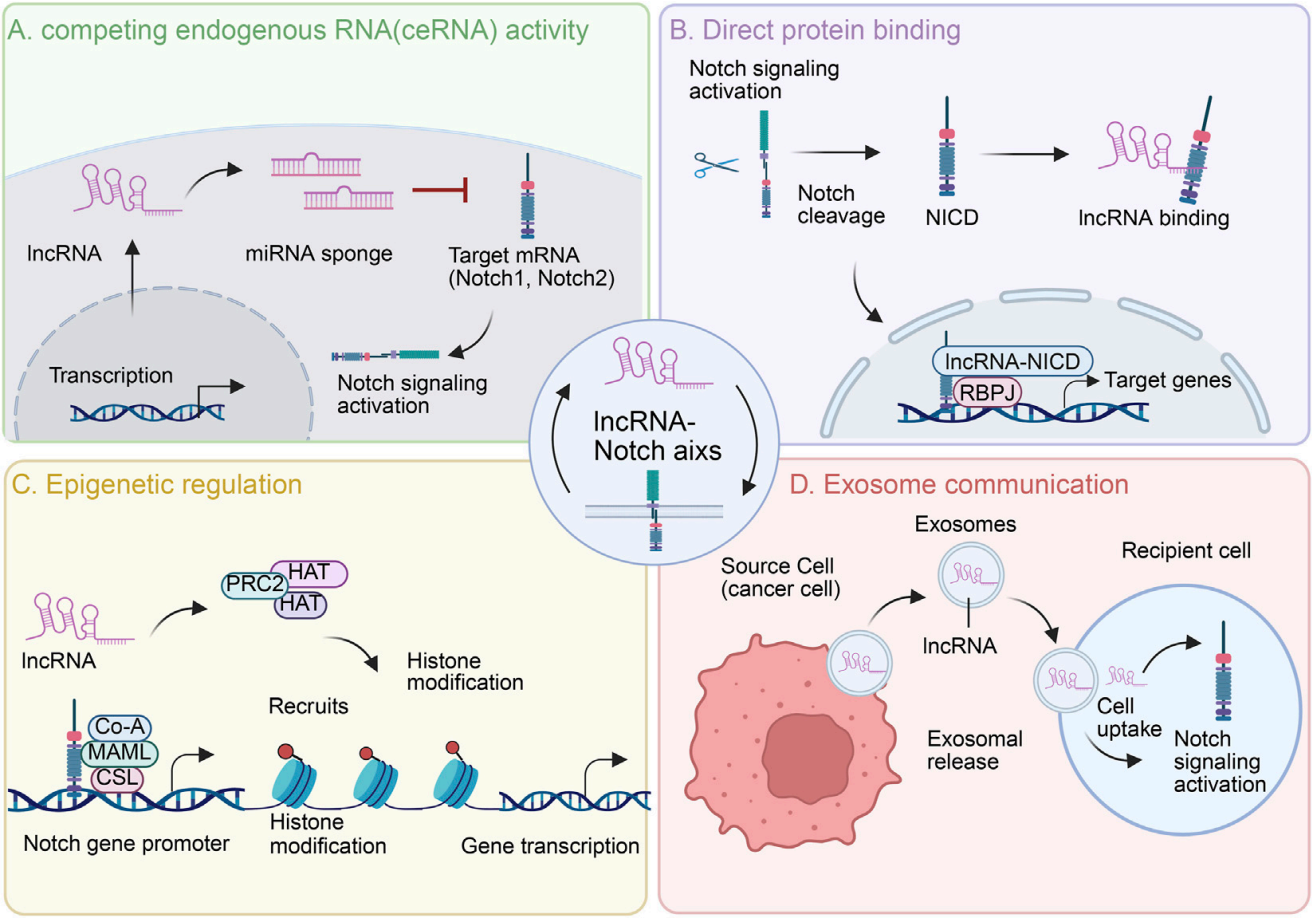
Illustration of four regulatory mechanisms of the lncRNA-Notch axis in cancers: **(A)** Competing endogenous RNA (ceRNA) activity. In the cytoplasm, specific lncRNAs act as miRNA sponges to sequester target miRNAs, thereby relieving the post-transcriptional repression on core Notch receptors (such as Notch1 and Notch2) or ligands, which leads to the activation of downstream Notch signaling; **(B)** Direct protein binding. Translocated or cytoplasmic lncRNAs physically interact with Notch signaling components, such as directly binding to the cleaved Notch intracellular domain (NICD) or complexing with RBPJ to enhance or stabilize downstream transcriptional complex formation; **(C)** Epigenetic regulation. LncRNAs recruit chromatin-remodeling complexes to the promoter regions of Notch pathway genes, orchestrating local histone modifications to modulate gene transcription; **(D)** Exosome communication. Oncogenic lncRNAs packaged within source cancer cell-derived exosomes are released into the extracellular space and taken up by neighboring recipient cells, activating local Notch signaling to mediate intercellular crosstalk within the tumor microenvironment (Figure created using BioRender.com). ceRNA competing endogenous RNA, lncRNA long non-coding RNA, miRNA microRNA, mRNA messenger RNA, NICD Notch intracellular domain, PRC2 polycomb repressive complex 2, HAT histone acetyltransferase, Co-A coactivator, MAML mastermind-like, CSL CBF1/suppressor of hairless/Lag1, RBPJ recombination signal binding protein for immunoglobulin kappa J region.

Emerging evidence underscores that lncRNAs and the Notch signaling pathway engage in extensive bidirectional crosstalk, critically influencing tumorigenesis and malignant progression across diverse cancer types (Figure 1). LncRNAs modulate Notch pathway activity through multifaceted mechanisms, most notably as ceRNAs that sequester miRNAs to derepress Notch components, and via direct interactions that stabilize the NICD. Conversely, Notch signaling can transcriptionally regulate or influence the expression of specific lncRNAs, establishing complex feedback loops. This aberrant interplay disrupts fundamental cellular processes, including cell proliferation, apoptosis, invasion, and chemoresistance. Meanwhile, an increasing number of studies have shown that this axis also has significant regulatory significance in clinical applications (Table 1). This section will elaborate on the specific roles of this regulatory axis in various cancers based on four mechanisms: ceRNA, protein binding, epigenetic regulation, and exosome communication (Figure 2).

**TABLE 1:** Mechanisms and clinical significance of lncRNA-mediated Notch pathway regulation in tumors.

Cancer type	LncRNA	Role	Mechanism	Evidence strength	Clinical feature	Therapeutic tractability	References
Tongue cancer	PTTG3P	Oncogene	Acts as a ceRNA to sponge miR-142-5p, upregulating the ligand JAG1 and EMT process	*In vitro* + clinical cohort (+ rescue assay)	—	Prognostic biomarker candidate; potential therapeutic target via siRNA silencing	Zhang et al. (2022)
Tongue cancer	UCA1	Oncogene	Acts as a ceRNA to sponge miR-124, thereby upregulating the expression of its target JAG1	*In vitro* + clinical cohort (+ rescue assay)	Poor prognosis	Prognostic biomarker candidate; potential therapeutic target via siRNA silencing	Zhang T. H. et al. (2019)
Laryngeal Cancer	PCAT6	Oncogene	Acts as a ceRNA to sponge miR-4731-5p, leading to the upregulation of NOTCH3	*In vitro* only (+ rescue assay)	—	Potential therapeutic target via shRNA silencing	Bai et al. (2024)
Laryngeal cancer	AFAP1-AS1	Oncogene	Acts as a ceRNA to sponge miR-320a, thereby upregulating​ the key Notch transcription factor RBPJ	*In vitro* + clinical cohort (+ rescue assay)	—	Prognostic biomarker candidate; potential target to reverse cisplatin chemoresistance	Yuan et al. (2018)
Tongue cancer	HOTAIR	Oncogene	Positively regulates Jagged-1/Notch-1 signaling and induces EMT	*In vitro* only	—	Potential therapeutic target via siRNA silencing	Zhang et al. (2021f)
Oral squamous cell carcinoma	HNF1A-AS1	Oncogene	The upregulation of HNF1A-AS1 induced by the transcription factor STAT3 activates the Notch signaling pathway	*In vitro* + clinical cohort (+ rescue assay)	Advanced T stage, nodal invasion, poor differentiation and poor overall survival	Prognostic biomarker candidate; potential therapeutic target via shRNA silencing	Liu et al. (2019)
Laryngeal squamous cell carcinoma	AC068768.1	Oncogene	Acts as a ceRNA to sponge miR-139-5p, leading to the upregulation of NOTCH1	*In vitro* + *in vivo* (+ rescue assay)	—	Exosomal liquid biopsy biomarker candidate; potential therapeutic target via shRNA silencing	Chen et al. (2024)
Oral squamous cell carcinoma	LINC01355	Oncogene	Upregulates Notch signaling pathway to inhibit CD8^+^ T cell anti-tumor activity and promote OSCC malignancy	*In vitro* + *in vivo* (+ rescue assay)	—	Potential immunotherapeutic target via shRNA silencing	Zou et al. (2021)
Oral squamous cell carcinoma	NEAT1	Oncogene	Upregulates VEGF-A​ and activates the Notch signaling pathway​ (Notch1, Notch2, Jagged)	*In vitro* only (+ rescue assay)	—	Potential therapeutic target combined with Notch inhibitor	He K. et al. (2020)
Gastric cancer	LINC00346	Oncogene	Sponges miR-34a-5p to upregulate NOTCH1, CD44, and AXL; activated by KLF5/MYC	*In vitro* + *in vivo* + clinical cohort (+ rescue assay)	Poor pathologic stage, larger tumor size, and poor prognosis	Diagnostic and prognostic biomarker candidate; potential therapeutic target via shRNA silencing	Xu et al. (2019)
Gastric cancer	DLEU2	Oncogene	STAT1-activated DLEU2 upregulates NOTCH2 by sponging miR-23b-3p, leading to activate Notch signaling	*In vitro* only (+ rescue assay)	—	Potential prognostic biomarker candidate; therapeutic target via silencing to block NOTCH2 activation	Li et al. (2021b)
Gastric cancer	SNHG22	Oncogene	Recruits EZH2 to repress tumor suppressor genes and sponges miR-200c-3p to upregulate Notch1 expression	*In vitro* + *in vivo* + clinical cohort (+ rescue assay)	Poor prognosis	Diagnostic/prognostic biomarker candidate	Mao et al. (2021)
Colorectal cancer	DSCAM-AS1	Oncogene	Acts as a ceRNA to sponge miR-137 and upregulate NOTCH1	*In vitro* + clinical cohort (+ rescue assay)	Tumor metastasis, advanced stage, and poor prognosis	Prognostic biomarker candidate; potential target via silencing NOTCH1 pathway	Xu et al. (2020b)
Colorectal cancer	LINC00707	Oncogene	Sponges miR-206 to upregulate NOTCH3 expression	*In vitro* + clinical cohort	Larger tumor size, lymphatic metastasis, and distant metastasis	Potential therapeutic target via silencing NOTCH3 pathway	Zhu et al. (2019)
Colorectal cancer	LINC01315	Oncogene	Sponges miR-484 to upregulate DLK1	*In vitro* + *in vivo* + clinical cohort (+ rescue assay)	—	Potential target via silencing to inhibit stemness and Notch pathway	Li Y. et al. (2022)
Gastric cancer	NALT1	Oncogene	Cis-regulates NOTCH1​ to activate Notch signaling	*In vitro* + clinical cohort (+ rescue assay)	Poor prognosis, tumor grade and survival	Prognostic biomarker candidate; potential target via inhibiting NOTCH pathway	Piao et al. (2019)
Colorectal cancer	LUNAR1	Oncogene	Notch induces LUNAR1 to activate the IGF1 pathway	*In vitro* + clinical cohort (+ rescue assay)	Tumor aggressiveness, shorter disease-free and overall survival	Prognostic biomarker candidate; potential target via silencing IGF1 pathway	Zhang Z. et al. (2019)
Hepatocellular carcinoma	lincRNA-p21	Tumor suppressor	Overexpression inhibits the Notch signaling pathway and EMT	*In vitro* + *in vivo* + clinical cohort (+ rescue assay)	—	Potential suppressor target via Notch pathway	Jia et al. (2016)
Hepatocellular carcinoma	ILF3-AS1	Oncogene	Sponges miR-628-5p to upregulate MEIS2 and elevates key proteins of the Notch pathway	*In vitro* + *in vivo* + rescue assay	—	Potential target via inhibiting Notch pathway	Yan et al. (2022)
Gallbladder cancer	TRPM2-AS	Oncogene	TRPM2-AS inhibits NUMB expression, thereby activating the NOTCH1 signaling pathway	*In vitro* + *in vivo* + clinical cohort + rescue assay	Tumor angiogenesis and poor prognosis	Promising angiogenic biomarker	He et al. (2024)
Cholangiocarcinoma	HANR	Oncogene	Binds to NICD and positively regulates Notch signaling by modulating RBP-Jκ transcriptional activity	*In vitro* only (+ rescue assay)	—	Potential therapeutic target	Zhou et al. (2023)
Gastric cancer	MIR4435-2HG	Oncogene	Induces M2 polarization by activating the Jagged1/Notch	*In vitro* + clinical cohort (+ rescue assay)	—	Exosomal liquid biopsy biomarker candidate; potential therapeutic target via siRNA silencing	Li C. et al. (2022)
Hepatocellular carcinoma	Linc00974	Oncogene	Undergoes promoter hypomethylation to become upregulated, then acts as a ceRNA to sponge miR-642, thereby activating the Notch and TGF-β signaling pathways	*In vitro* + *in vivo* (+ rescue assay)	Larger tumor size, poorer differentiation, advanced TNM stage, and metastasis	Prognostic biomarker candidate; Potential therapeutic target (siRNA-compatible)	Tang et al. (2014)
Pancreatic cancer	MIR99AHG	Oncogene	Sponges miR-3129-5p and recruits ELAVL1 to upregulate NOTCH2	*In vitro* + *in vivo* (+ rescue assay)	—	Potential therapeutic target (siRNA-compatible)	Xu et al. (2021)
Pancreatic cancer	SNHG7	Oncogene	Induced by mesenchymal stem cells and directly interacts with Notch1 to activate the Notch1/Jagged1/Hes-1 signaling pathway	*In vitro* + *in vivo* (+ rescue assay)	—	Prognostic biomarker candidate; Potential therapeutic target	Cheng D. et al. (2021)
Lung adenocarcinoma	LINC01123	Oncogene	Transcriptional activation by ZEB1 drives LINC01123 to sponge miR-449b-5p, leading to NOTCH1 upregulation	*In vitro* only (+ rescue assay)	—	Potential therapeutic target	Zhang M. et al. (2020)
Non-small cell lung cancer	LINC01806	Oncogene	Transcriptional activation by STAT1 enables it to sponge miR-4428 and upregulate NOTCH2	*In vitro* + *in vivo* (+ rescue assay)	—	Potential therapeutic target (siRNA-compatible)	Huang et al. (2022)
Non-small cell lung cancer	LINC01783	Oncogene	Sponges miR-432-5p to upregulate the Notch ligand DLL1	*In vitro* + *in vivo* (+ rescue assay)	—	Potential therapeutic target (siRNA-compatible)	Deng et al. (2021)
Non-small cell lung cancer	KCNQ1OT1	Oncogene	Sponges miR-129-5p to upregulate the expression of JAG1	*In vitro* + clinical cohort (+ rescue assay)	Shorter overall survival, advanced tumor stage, and lymph node metastasis	Prognostic biomarker candidate	Wang Y. et al. (2020)
Lung adenocarcinoma	TUSC7	Tumor suppressor	Sponges miR-146 to upregulate NUMB, thereby inactivating the NOTCH signaling pathway	*In vitro* only (+ rescue assay)	—	—	Huang et al. (2019)
Lung cancer	AGAP2-AS1	Oncogene	Exosomal lncRNA sponges miR-296 to upregulate NOTCH2	*In vitro* + *in vivo* + clinical cohort (+ rescue assay)	Radioresistance and serves as a prognostic predictor	Prognostic biomarker candidate; Potential therapeutic target (siRNA-compatible); Exosomal liquid biopsy biomarker candidate	Zhang et al. (2021a)
Non-small cell lung cancer	FEZF1-AS1	Oncogene	Sponges miR-34a to upregulate NOTCH1 expression	*In vitro* + clinical cohort (+ rescue assay)	Advanced clinical stages	Prognostic biomarker candidate	Huang S. et al. (2020)
Cervical cancer	DARS-AS1	Oncogene	Sponges miR-628-5p to upregulate JAG1 expression	*In vitro* only (+ rescue assay)	—	Potential therapeutic target	Chen et al. (2020)
Breast cancer	SNHG3	Oncogene	Sponges miR-154-3p to upregulate Notch2 expression	*In vitro* + *in vivo* (+ rescue assay)	—	Potential therapeutic target (siRNA-compatible)	Jiang et al. (2020)
Breast cancer	SNHG7	Oncogene	Sponges miR-34a to activate the Notch-1 signaling pathway	*In vitro* + *in vivo* (+ rescue assay)	—	Potential therapeutic target (siRNA-compatible)	Sun et al. (2019)
Triple-negative breast cancer	CCAT2	Oncogene	Promotes stemness by upregulating OCT4-PG1 and sponging miR-205 to activate the Notch pathway	*In vitro* + *in vivo* (+ rescue assay)	—	Potential therapeutic target (siRNA-compatible)	Xu et al. (2020c)
Cervical cancer	HOXA-AS2	Oncogene	Directly binds to NICD to enhance its interaction with RBP-JK	*In vitro* only (+ rescue assay)	—	Potential therapeutic target	Wu Q. et al. (2021)
Endometrial cancer	SNHG12	Oncogene	SNHG12 expression upregulated by ZIC2, and activates the Notch signaling pathway	*In vitro* only (+ rescue assay)	—	—	Cai et al. (2021)
Ovarian cancer	TUG1	Oncogene	Sponges miR-1299 to upregulate NOTCH3 expression and NOTCH3 in turn upregulates TUG1	*In vitro* + *in vivo* + clinical cohort (+ rescue assay)	—	Prognostic biomarker candidate; Potential therapeutic target	Pei et al. (2020)
Breast cancer	linc-OIP5	Oncogene	Upregulates JAG1 in cancer cells to activate the Notch pathway in endothelial cells, promoting angiogenesis	*In vitro* only (+ rescue assay)	Tumor grade	Prognostic biomarker candidate	Zhu et al. (2020)
Bladder cancer	LINC00960, LINC02470	Oncogene	Delivered by exosomes to activate the Notch signaling pathway	*In vitro* only (+ rescue assay)	Potential liquid biomarkers for prognostic surveillance	Exosomal liquid biopsy biomarker candidate	Huang C. S. et al. (2020)
Renal cell carcinoma	LINC01939	Tumor suppressor	Downregulates miR-154 to inactivate Notch signaling pathways	*In vitro* only (+ rescue assay)	—	—	Zhang R. et al. (2020)
Endometrial carcinoma	MEG3	Tumor suppressor	Overexpressed MEG3 inhibits the expression of Notch1 and Hes1, thereby suppressing the Notch pathway	*In vitro* + *in vivo* (+ rescue assay)	—	—	Guo et al. (2016)
Ovarian cancer	SRA	Oncogene	Activates Notch signaling​ and EMT	*In vitro* + *in vivo* + clinical cohort	Poor prognosis	Prognostic biomarker candidate; Potential therapeutic target	Kim et al. (2021)
Triple-negative breast cancer	MALAT1	Oncogene	Packaged into exosomes; sponges miR-140-5p to upregulate JAG1​ and VEGFA	*In vitro* + *in vivo* (+ rescue assay)	Poor prognostic	Exosomal liquid biopsy biomarker candidate; Potential therapeutic targe	Liu et al. (2023)
Breast cancer	linc00514	Oncogene	Transcriptionally upregulates Jagged1 expression by enhancing STAT3 phosphorylation	*In vitro* + *in vivo* (+ rescue assay)	—	Prognostic biomarker candidate; Potential therapeutic target	Tao et al. (2020)
Cervical cancer	GABPB1-AS1	Oncogene	Sponges miR-519e-5p to upregulate the expression of the oncogene NOTCH2	*In vitro* + *in vivo* + clinical cohort (+ rescue assay)	Tumor size and lymph node metastasis	Prognostic biomarker candidate; Potential therapeutic target (siRNA-compatible)	Ou et al. (2020)
T-ALL	LUNAR1	Oncogene	Transcriptionally activated by oncogenic Notch1, and enhances IGF1R expression	*In vitro* + *in vivo* (+ rescue assay)	—	Potential therapeutic target (ASO-compatible)	Trimarchi et al. (2014)
T-ALL	NALT	Oncogene	Activates the NOTCH1 signaling pathway through cis-regulation of its adjacent NOTCH1 gene	*In vitro* + *in vivo* (+ rescue assay)	—	Potential therapeutic target (ASO-compatible)	Wang et al. (2015)
T-ALL	PPM1A-AS	Oncogene	Simultaneously upregulating Notch4, STAT3, and Akt signaling pathway	*In vitro* + *in vivo* (+ rescue assay)	—	Potential therapeutic target (siRNA-compatible)	Li et al. (2021a)
Multiple Myeloma	MALAT1	Oncogene	MiR-125b regulated MALAT1 expression via Notch1 signaling pathway	*In vitro* + *in vivo* (+ rescue assay)	—	Potential therapeutic target (miR-125b mimic-compatible	Gao et al. (2018)
Glioma	FOXD2-AS1	Oncogene	Recruits TAF-1​ to the NOTCH1​ promoter, activating Notch signaling	*In vitro* + *in vivo* + clinical cohort (+ rescue assay)	A novel therapeutic candidate	Prognostic biomarker candidate; Potential therapeutic target (siRNA-compatible)	Wang Y. et al. (2022)
Glioma	PlncRNA-1	Oncogene	Activates Notch signaling (NOTCH1/JAG1/HES1)	*In vitro* + clinical cohort (+ rescue assay)	Overall survival and progression-free survival	Prognostic biomarker candidate	Wang X. et al. (2018)
Glioma	ZFAS1	Oncogene	Activates Notch signaling​ and EMT	*In vitro* + clinical cohort (+ rescue assay)	Tumor stage and poor overall survival	Prognostic biomarker candidate	Gao et al. (2017)
Glioblastoma	LINC01152	Oncogene	LINC01152 upregulates MAML2 through the miR-466/SRSF1 axis, forms the RBPJ/MAML2 complex and feedback-activates its own transcription	*In vitro* + *in vivo* (+ rescue assay)	—	Prognostic biomarker candidate	Wu J. et al. (2021)
Glioma	HOXA-AS2	Oncogene	Sponges miR-302a to upregulate KDM2A, leading to increased expression of the Notch ligand JAG1	*In vitro* + *in vivo* (+ rescue assay)	—	Prognostic biomarker candidate; Potential therapeutic target (siRNA-compatible)	Zhong et al. (2022)
Glioblastoma	RAMP2-AS1	Tumor suppressor	Directly binds to DHC10 protein, leading to indirect inhibition of NOTCH3 expression	*In vitro* + *in vivo* + clinical cohort (+ rescue assay)	—	Prognostic biomarker candidate	Liu et al. (2016)
Glioblastoma	FEZF1-AS1	Oncogene	Sponge miR-34a to upregulate Notch1	*In vitro* + clinical cohort (+ rescue assay)	Poor survival	Prognostic biomarker candidate	Luo et al. (2020)
Retinoblastoma	PVT1	Oncogene	Sponges miR-488-3p to upregulate NOTCH2	*In vitro* + *in vivo* + clinical cohort (+ rescue assay)	Poor overall survival	Prognostic biomarker candidate; Potential therapeutic target (siRNA-compatible)	Wu X. Z. et al. (2019)
Retinoblastoma	TUG1	Oncogene	Activates Notch signaling​ and EMT	*In vitro* only (+ rescue assay)	Poor prognosis	—	Wang H. et al. (2022)
Melanoma	BANCR	Oncogene	Sponges miR-204 to upregulate Notch2	*In vitro* + *in vivo* (+ rescue assay)	—	Potential therapeutic target (siRNA-compatible)	Cai et al. (2017)
Cutaneous Malignant Melanoma	ZFPM2-AS1	Oncogene	Sponges miR-650 to upregulate NOTCH1	*In vitro* only (+ rescue assay)	—	Potential therapeutic target	Liu et al. (2021)
Osteosarcoma	CRNDE	Oncogene	Activates Notch1/JAG1​ signaling and EMT	*In vitro* + *in vivo* + clinical cohort (+ rescue assay)	Poor prognosis	Prognostic biomarker candidate; Potential therapeutic target (siRNA-compatible)	Li et al. (2018)
Osteosarcoma	SNHG12	Oncogene	Sponges miR-195-5p to upregulate Notch2	*In vitro* + clinical cohort (+ rescue assay)	Poor prognosis	Prognostic biomarker candidate; Potential therapeutic target	Zhou et al. (2018)
Hemangioma	MEG8	Oncogene	Knockdown upregulates miR-203, leading to downregulation of JAG1 and Notch1, thereby inhibiting the Notch signaling	*In vitro* only (+ rescue assay)	—	—	Hu et al. (2021)
Hemangioma	MEG8	Oncogene	Sponges miR-497-5p to upregulate NOTCH2, leading to increased expression of the ferroptosis inhibitors SLC7A11 and GPX4	*In vitro* only (+ rescue assay)	—	—	Ma et al. (2021)

Although ceRNA-based regulation is the most frequently reported mode in the current literature, this frequency should not be equated with exclusive or superior biological importance. Therefore, the following sections distinguish reported frequency from mechanistic significance and emphasize both ceRNA and non-ceRNA mechanisms. To accurately evaluate the biological relevance of these axes, rigorous mechanistic validation standards—comprising loss/gain-of-function, functional rescues, and translational corroboration—must be carefully weighed (De Blasio et al., 2019).

### 4.1 Head and neck tumors

LncRNAs critically regulate the Notch signaling pathway through diverse mechanisms, significantly influencing tumor progression in head and neck cancers, particularly oral and laryngeal squamous cell carcinomas (OSCC/LSCC). First, ceRNA interaction is the most prevalent regulatory mode in head and neck cancers. For instance, in tongue cancer, lncRNA PTTG3P sponges miR-142-5p to upregulate the Notch ligand JAG1, thereby activating the pathway and promoting cancer aggressiveness (Zhang et al., 2022). Furthermore, a similar ceRNA axis is observed where lncRNA UCA1 sequesters miR-124, leading to the derepression of JAG1 and subsequent activation of Notch signaling to drive EMT and invasion; interestingly, UCA1 converges with another lncRNA, MALAT1, on this same miR-124/JAG1 axis, highlighting a complex regulatory crosstalk (Zhang T. H. et al., 2019). Additionally, in laryngeal carcinoma, lncRNA PCAT6 functions as a sponge for miR-4731-5p, resulting in the upregulation of Notch3 and enhanced tumor cell proliferation (Bai et al., 2024). AFAP1-AS1 promotes tumor stemness and chemoresistance by sequestering miR-320a, thereby increasing the expression of the core transcription factor RBPJ (Yuan et al., 2018).

Moreover, lncRNA HOTAIR promotes tongue cancer progression by positively regulating the protein levels of key Notch pathway members, including JAG1, Notch1, and the downstream effector Hes1, although the precise molecular interaction remains to be fully elucidated (Zhang et al., 2021f). Furthermore, the oncogenic role of lncRNA HNF1A-AS1 in OSCC is linked to its STAT3-induced overexpression, which in turn activates the Notch pathway (Liu et al., 2019). Apart from cell-autonomous mechanisms, lncRNAs also modulate the tumor microenvironment. A striking example is lncRNA AC068768.1, which is secreted by LSCC cells via exosomes. This exosomal AC0687681 is taken up by tumor-associated macrophages, inducing their M2 polarization, and also acts within cancer cells as a ceRNA for miR-139-5p to upregulate Notch1, creating a Notch1/STAT3 positive feedback loop that drives metastasis (Chen et al., 2024).

Interestingly, the interaction between lncRNAs and Notch signaling also plays a pivotal role in regulating anti-tumor immunity. In OSCC, lncRNA LINC01355 promotes immune evasion by enhancing Notch signaling, which subsequently suppresses the anti-tumor activity of CD8^+^ T cells; silencing LINC01355 inhibits tumor growth by reactivating CD8^+^ T cell function through Notch pathway inactivation (Zou et al., 2021). Furthermore, bioinformatic analyses have identified a prognostic 4-lncRNA signature in laryngeal cancer, which is predicted to influence patient outcomes potentially through regulation of the Notch pathway, among other signaling cascades (Zhang G. et al., 2019). Finally, the regulatory network can involve other pathways, as seen with lncRNA NEAT1, which promotes OSCC progression in conjunction with VEGF-A, with its oncogenic effects significantly dependent on downstream Notch signaling activation (He K. et al., 2020).

In conclusion, in head and neck tumors, the lncRNA-Notch axis is characterized by a receptor- and ligand-centered ceRNA layer, especially involving JAG1, Notch1, Notch3, and RBPJ. Non-ceRNA mechanisms are also evident through HOTAIR-associated pathway activation, STAT3-linked transcriptional regulation, exosomal AC068768.1 transfer, and immune reprogramming mediated by LINC01355. Thus, ceRNA regulation is the most visible pattern, but direct pathway modulation and tumor-immune communication may be equally important for invasion, metastasis, and immune escape.

### 4.2 Digestive system tumors

**FIGURE 3:**
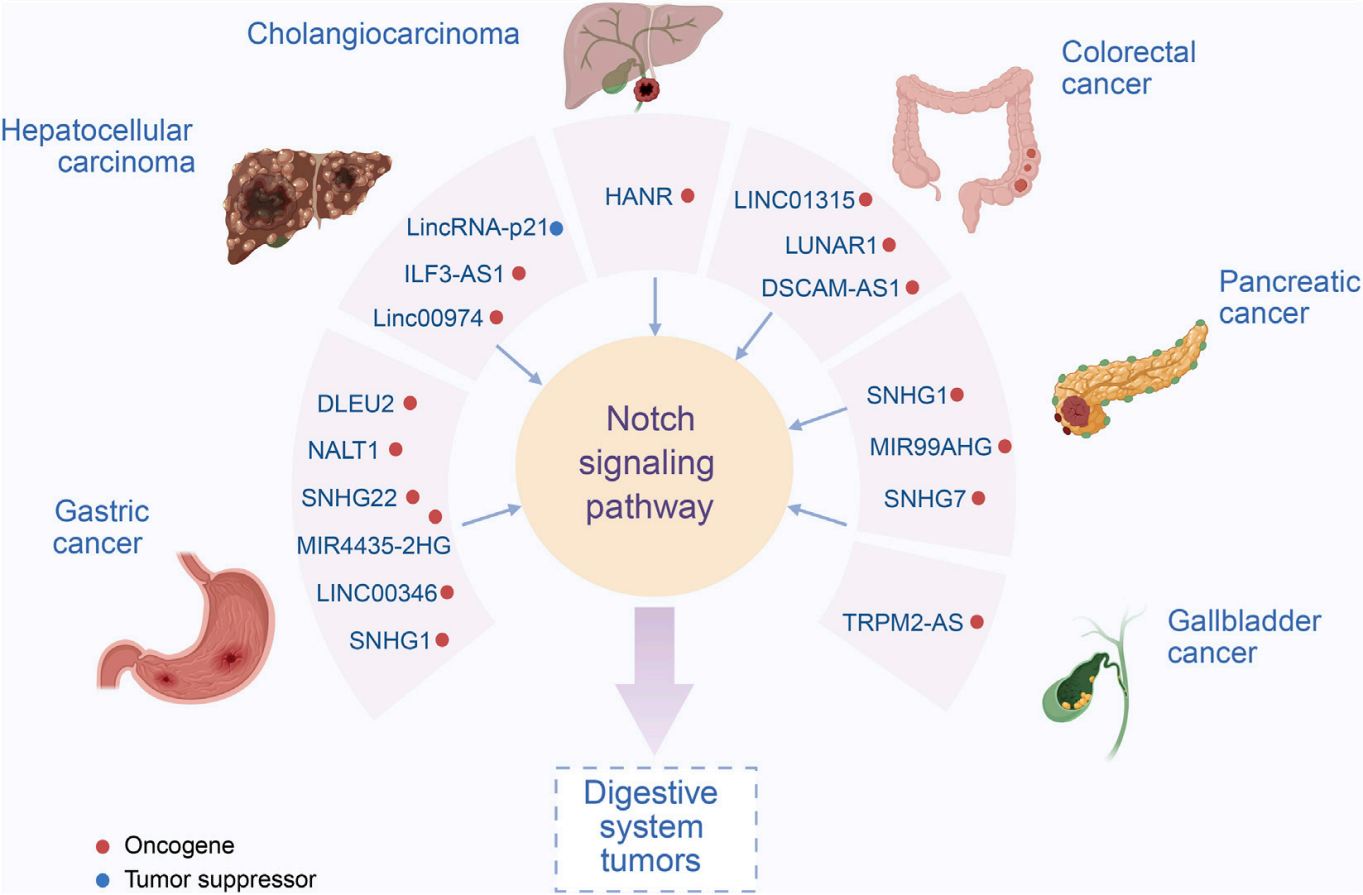
Overview of the interaction of lncRNA and the Notch signaling pathway in gastrointestinal tumors. Different lncRNAs aberrantally regulate the Notch signaling cascade across five major digestive system malignancies, encompassing gastric cancer, colorectal cancer, liver cancer, gallbladder cancer, and pancreatic cancer, where blue nodes/arrows represent tumor-suppressive components and red nodes/arrows represent oncogenic components (Figure created using BioRender.com).

In digestive system tumors, lncRNAs and the Notch signaling pathway form a complex regulatory axis (Figure 3) (Pan et al., 2018). The ceRNA mechanism plays a crucial regulatory role in gastrointestinal malignancies. At the core mechanistic level, several lncRNAs function through defined molecular pathways. LINC00346 acts as a molecular sponge for miR-34a-5p, co-upregulating oncogenes like Notch1 (Xu et al., 2019). Furthermore, DLEU2, transcriptionally activated by STAT1, drives tumor progression via the miR-23b-3p/Notch2 axis (Li et al., 2021b). Apart from that, SNHG1 promotes EMT by suppressing miR-15b, leading to the upregulation of DCLK1 and subsequent indirect activation of Notch1 signaling (Liu et al., 2020). These fundamental discoveries are directly supported by clinical evidence. In the original cohort study involving 60 paired gastric cancer tissues, elevated expression of SNHG22 was observed in 44 tumor samples ( p<0.0001 ) and was demonstrated to be significantly associated with poor overall survival of patients ( p=0.0007 ) (Mao et al., 2021).

Research in colorectal cancer demonstrates the broad functional scope of the lncRNA-Notch axis, ranging from cell-autonomous mechanisms to microenvironmental communication. A series of studies have elucidated key intracellular mechanisms. DSCAM-AS1 and LINC00707 specifically target the Notch1 and Notch3 receptors by sponging miR-137 and miR-206, respectively (Xu et al., 2020b; Zhu et al., 2019); LINC01315 influences cancer stem cell traits by upregulating the non-canonical Notch ligand DLK1 via miR-484 (Li Y. et al., 2022), and NALT1 is predicted to affect Notch1 expression through cis-regulation (Piao et al., 2019). Notably, this axis also mediates intercellular “crosstalk”; for instance, LUNAR1, directly activated by Notch signaling, drives colorectal cancer progression via IGF1 pathway activation, creating a positive feedback loop (Zhang Z. et al., 2019).

The functional modes of the lncRNA-Notch axis are remarkably diverse in HCC and biliary tract cancers. In HCC, its role is dualistic: the tumor suppressor lincRNA-p21 inhibits EMT by negatively regulating the Notch pathway (Jia et al., 2016), whereas the oncogenic ILF3-AS1 indirectly stabilizes MEIS1 and activates Notch signaling by competitively binding to miR-628-5p against MEIS2 mRNA (Yan et al., 2022). In biliary tract cancers, studies reveal more direct interactions: in gallbladder cancer (GBC), TRPM2-AS promotes angiogenesis by activating Notch1 signaling in endothelial cells via an exosome-mediated pathway (He et al., 2024). Meanwhile, in intrahepatic cholangiocarcinoma, HANR enhances the transcriptional activity of Notch by directly binding to the NICD (Zhou et al., 2023).

Recent findings further expand the functional scope of this axis. LncRNA MIR4435-2HG is delivered via gastric cancer cell-derived exosomes to macrophages, inducing M2 polarization by activating the Jagged1/Notch pathway, thereby shaping an immunosuppressive microenvironment (Li C. et al., 2022). Additionally, the stable fragment Linc00974F-1, detectable in plasma, shows promise as a non-invasive diagnostic biomarker when combined with CYFRA21-1 (Tang et al., 2014).

In pancreatic cancer, emerging evidence underscores the pivotal role of the lncRNA-Notch axis in driving tumor progression and therapy resistance. LncRNA MIR99AHG, induced by FOXA1, exerts its oncogenic function through a dual mechanism: sponging miR-3129-5p and recruiting ELAVL1 to stabilize NOTCH2 mRNA, thereby amplifying Notch signaling (Xu et al., 2021). Furthermore, the tumor microenvironment plays a critical role in modulating this axis. Mesenchymal stem cells can upregulate lncRNA SNHG7 in pancreatic cancer cells, which interacts with Notch1 and activates the Notch1/Jagged1/Hes-1 signaling cascade. This MSC-induced SNHG7/Notch1 interaction is a key mechanism conferring cancer stemness and resistance to the chemotherapeutic regimen Folfirinox (Cheng D. et al., 2021).

Digestive system tumors show both shared and tissue-specific regulatory patterns. Gastric and colorectal cancers are enriched for ceRNA and feedback-loop mechanisms that connect Notch receptors or ligands with EMT, stemness, and biomarker potential. In contrast, hepatobiliary and pancreatic tumors more prominently illustrate non-ceRNA regulation, including NICD binding, exosome-mediated endothelial or macrophage reprogramming, and RNA-binding-protein-dependent stabilization of NOTCH2 mRNA. These differences may reflect anatomical exposure to microbiota and inflammation, endoderm-derived epithelial programs, and the stromal richness of pancreatic and biliary tumors. However, whether these tissue contexts causally determine the dominant lncRNA-Notch regulatory mode remains unresolved.

### 4.3 Respiratory system tumors

In respiratory system tumors, primarily non-small cell lung cancer (NSCLC) and lung adenocarcinoma (LUAD), lncRNAs interact with the Notch signaling pathway through a sophisticated, multi-layered regulatory network that profoundly influences tumorigenesis, therapeutic resistance, and immune microenvironment remodeling. The most prevalent mechanism involves lncRNAs functioning as ceRNAs. For instance, the lncRNA LINC01123 is transcriptionally activated by the EMT-inducing factor ZEB1. It acts as a molecular sponge for miR-449b-5p, as demonstrated by dual-luciferase reporter and RNA pull-down assays, thereby relieving miR-449b-5p-mediated repression of its target gene NOTCH1. This enhances Notch signaling pathway activity and promotes malignant phenotypes in lung cancer cells (Zhang M. et al., 2020). Similarly, SNHG15 expression is induced by the transcription factor STAT1. In gefitinib-resistant LUAD cells, it competitively binds to miR-451, alleviating its suppression on Notch1 and contributing significantly to acquired resistance to tyrosine kinase inhibitors (Huang J. et al., 2020). Another STAT1-induced lncRNA, LINC01806, was confirmed through dual-luciferase reporter assays, RNA pull-down, and RIP experiments to act as a molecular sponge for miR-4428. It upregulates the target gene NOTCH2 and activates the Notch signaling pathway, thereby promoting proliferation, migration, and invasion of non-small cell lung cancer cells (Huang et al., 2022). Beyond Notch receptors, this ceRNA network extends to pathway ligands. The lncRNA LINC01783 enhances the expression of the ligand DLL1 by sponging miR-432-5p, thereby activating the pathway (Deng et al., 2021), while KCNQ1OT1, which is associated with poor patient prognosis, upregulates the ligand JAG1 by sequestering miR-129-5p (Wang Y. et al., 2020). The tumor-suppressive lncRNA TUSC7 presents a contrasting mechanism; it inhibits cancer stem cell renewal by sponging miR-146a, which stabilizes the Notch pathway inhibitor NUMB, leading to subsequent pathway suppression (Huang et al., 2019). The regulatory scope extends beyond cell-autonomous mechanisms to intercellular communication. Specifically, M2 macrophage-derived exosomes deliver the lncRNA AGAP2-AS1 to tumor cells, where it functions as a ceRNA for miR-296, upregulating Notch2 and promoting a radioresistant phenotype (Zhang et al., 2021a). Furthermore, environmental cues are integral to this network. Chronic exposure to the tobacco-specific carcinogen NNK suppresses EGFR-AS1 while activating the HIF2A/FOXP3 axis, ultimately driving Notch1 signaling and enhancing cancer stemness (Qi et al., 2019). Additionally, FEZF1-AS1 promotes cell invasion and migration by acting as a molecular sponge for miR-34a, leading to the activation of Notch1 (Huang S. et al., 2020).

In summary, respiratory tumors are marked by strong coupling between environmental induction and lncRNA-Notch activation. Smoking-related signals, EMT transcription factors, STAT1-driven lncRNA induction, and macrophage-derived exosomal transfer converge on Notch1, Notch2, DLL1, and JAG1. The frequent ceRNA axes in NSCLC and LUAD should therefore be interpreted within a broader network that includes transcriptional activation, radioresistance, tyrosine kinase inhibitor resistance, and microenvironmental remodeling.

### 4.4 Urogenital tumors

**FIGURE 4:**
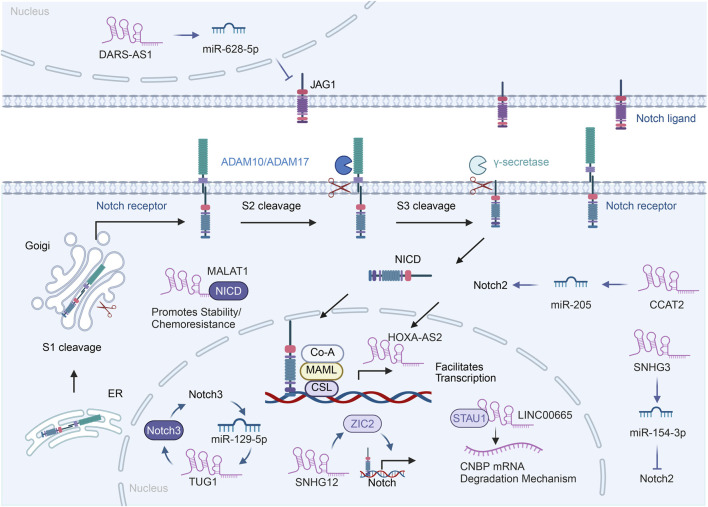
LncRNAs exert a critical influence by modulating the Notch signaling pathway in Urogenital tumors. The lncRNA-Notch signaling axis operates as a critical regulatory command hub in urogenital malignancies, driving key malignant phenotypes such as cell proliferation, invasion, EMT, cancer stemness, and therapy resistance. This diagram systematically illustrates the multi-layered molecular mechanisms—including upstream transcriptional activation, cytoplasmic ceRNA sponging, direct nuclear protein interactions, and alternative mRNA degradation pathways—through which various lncRNAs precisely fine-tune canonical Notch signaling activity (Figure created using BioRender.com). EMT epithelial-mesenchymal transition, ADAM10/ADAM17 a disintegrin and metalloprotease 10/17, NICD Notch intracellular domain, Co-A coactivator, MAML mastermind-like, CSL CBF1/suppressor of hairless/Lag1.

Extensive research has delineated a complex regulatory network between lncRNAs and the Notch signaling pathway in urogenital cancers (Figure 4), wherein the ceRNA model is a well-validated fundamental mechanism. A classic example is observed in cervical cancer (CC): DARS-AS1 sponges miR-628-5p, relieving its repression of the Notch ligand JAG1 and activating the oncogenic Notch signaling (Chen et al., 2020). This regulatory paradigm is also confirmed in breast cancer (BC), where SNHG3 and SNHG7 promote tumor progression by activating the Notch2 and Notch1 signaling pathways through sequestering miR-154-3p and miR-34a, respectively (Jiang et al., 2020; Sun et al., 2019). Furthermore, in triple-negative breast cancer (TNBC), CCAT2 enhances Notch2 expression by acting as a molecular sponge for miR-205, which in turn synergizes with OCT4-PG1 to enhance cancer stemness, revealing the critical role of the ceRNA mechanism in regulating cancer stem cell properties (Xu et al., 2020c).

Beyond the classic ceRNA mechanism, direct RNA-protein interactions add a layer of complexity to this regulatory axis. In OC, MALAT1 directly physically binds the NICD protein, a key step in promoting chemoresistance (Bai et al., 2018). Complementarily, in CC, HOXA-AS2 enhances the transcriptional activity of the Notch pathway by facilitating the formation of the NICD/RBP-JK transcriptional activation complex (Wu Q. et al., 2021). Additionally, transcriptional regulation and self-regulatory feedback loops introduce further dimensions of control. In endometrial cancer (EC), the transcription factor ZIC2 acts as an upstream activator of SNHG12 expression, which subsequently modulates the Notch pathway (Cai et al., 2021). A salient example of a positive feedback loop exists in OC, where Notch3 signaling transcriptionally upregulates TUG1 expression, and TUG1, in turn, reinforces Notch3 signaling by acting as a ceRNA for miR-129-5p, thereby establishing a self-amplifying oncogenic circuit (Pei et al., 2020). Notably, LINC00665 mediates the degradation of CNBP mRNA via its Alu elements and the STAU1 protein, indirectly affecting the Notch and Wnt pathways, showcasing a novel post-transcriptional regulatory mechanism independent of ceRNA (Liu et al., 2024). The functional outcome of Notch signaling is highly context-dependent. It primarily functions as an oncogene, as exemplified in BC where LINC01235 transcriptionally regulates NFIB to influence Notch signaling output, and where linc-OIP5 promotes tumor angiogenesis by coordinating a Hippo (YAP1)-Notch (JAG1) signaling circuit (Zhu et al., 2020). Similarly, in bladder cancer, exosome-derived lncRNAs LINC00960 and LINC02470 from high-grade cells promoteEMTand aggressiveness in recipient cells by activating Notch signaling alongside beta-catenin and Smad2/3 pathways (Huang C. S. et al., 2020). Beyond these lncRNA-mediated mechanisms, emerging evidence underscores the critical role of chronic inflammation as a potent driver of malignant transformation within the urogenital system. For instance, recent pioneering work demonstrated that the interstitial cystitis-related gene CCDC8 significantly promotes bladder cancer tumorigenesis by facilitating CUL7-mediated proteasomal degradation of P53 [New Ref]. This finding provides direct molecular evidence that extrinsic inflammatory signals can directly converge on core intrinsic tumor-suppressor pathways, successfully broadening the mechanistic landscape of urogenital oncogenesis (Wang J. et al., 2026). Conversely, in specific pathological contexts, Notch exhibits potent tumor-suppressive functions. In renal cell carcinoma (RCC), LINC01939 acts as a tumor suppressor by inhibiting the Notch pathway (Zhang R. et al., 2020). In EC, MEG3 suppresses tumor progression by negatively regulating Notch signaling (Guo et al., 2016). This remarkable mechanistic diversity underscores the complex and multilayered interactions between lncRNAs and the Notch pathway, contributing significantly to tumor heterogeneity.

Parallel to these mechanistic discoveries, the clinical significance of the lncRNA-Notch axis is increasingly recognized, offering great promise for advancing cancer diagnosis and therapy. Key components of this axis have become valuable prognostic biomarkers. In OC, high expression of SRA is an independent predictor of shorter overall survival, highlighting its clinical utility in risk stratification (Kim et al., 2021). In-depth studies on MALAT1 show that its high expression is significantly associated with poor patient prognosis in OC and is directly linked to cisplatin resistance, underscoring its clear clinical relevance (Liu et al., 2023).

A major clinical challenge in oncology is the development of therapy resistance, and the lncRNA-Notch axis is considered a core mediator of this process. DANCR-driven docetaxel resistance in PCa emphasize the pivotal role of this axis in treatment failure (Ma et al., 2019). These insights reveal its potential as a novel therapeutic target. For instance, the anti-tumor effect of anisomycin in OC is mediated, at least partially, through the downregulation of the MEG3/miR-421/PDGFRA axis and the consequent inhibition of downstream Notch signaling (Ye et al., 2019). Furthermore, the role of specific lncRNAs in reshaping the TME opens new avenues for combination therapies. The lncRNA linc00514 modulates the TME by activating the STAT3/Jagged1/Notch axis, leading to the polarization of tumor-associated macrophages towards the typically immunosuppressive M2 phenotype (Tao et al., 2020). Consequently, targeting this axis could potentiate the efficacy of immunotherapy.

Particularly important is the axis’s role in the etiology of virus-associated cancers. In HPV16-positive CC, the viral oncoprotein E6 induces the expression of GABPB1-AS1, which then sponges miR-579-3p to upregulate Notch1, directly linking the viral oncogenic factor to Notch pathway activation and providing new insights for targeted therapy in these cancers (Ou et al., 2020).

Urogenital tumors display pronounced context dependence. Cervical, breast, ovarian, prostate, bladder, renal, and endometrial cancers share recurrent ceRNA-mediated regulation of JAG1, Notch1, and Notch2, but their dominant biological outputs differ. Gynecologic and breast cancers often link the axis to stemness, angiogenesis, and chemoresistance, whereas bladder cancer emphasizes exosome-mediated EMT and renal or endometrial cancer can show tumor-suppressive Notch regulation. Viral exposure adds another layer in HPV-positive cervical cancer, where the E6-GABPB1-AS1-miR-579-3p-Notch1 cascade connects viral oncogenesis with Notch activation. These observations argue against a single universal model and highlight the need to define cell type, hormonal context, viral status, and therapy pressure in future studies.

### 4.5 Hematologic malignancies

Unlike the ceRNA-dominated model in solid tumors, in hematological malignancies, direct transcriptional activation and cis-epigenetic regulatory circuits between Notch and lncRNAs constitute the most dominant core mechanism. In T-cell acute lymphoblastic leukemia (T-ALL), the Notch signaling pathway orchestrates a complex oncogenic network through dynamic interactions with lncRNAs, operating at multiple regulatory levels. For example, the Notch1 transcriptional activation complex directly binds to the LUNAR1 promoter and induces its expression. This, in turn, interacts with the IGF1R enhancer through a chromatin loop confirmed by chromosome conformation capture (3C), maintaining IGF1R transcription and IGF1 signaling pathway activity, thereby driving tumor progression (Trimarchi et al., 2014). Additionally, NALT, located approximately 400 bp upstream of the NOTCH1 gene, acts as a transcriptional co-activator in cis, upregulating NOTCH1 transcription and forming a positive feedback loop, as demonstrated by the Gal4-λN/BoxB reporter system, further amplifying Notch signaling output and oncogenic drive (Wang et al., 2015). The complexity escalates to pathway crosstalk, where the lncRNA PPM1A-AS acts as a multi-pathway coregulator, simultaneously upregulating Notch4, STAT3, and Akt to coordinately activate several key oncogenic cascades (Li et al., 2021a). This network exhibits remarkable plasticity under therapeutic pressure; prolonged inhibition of the Wnt/beta-catenin pathway triggers a compensatory upregulation of Notch1/2 signaling, revealing a built-in escape mechanism that contributes to therapy resistance (Zhang et al., 2021d). Beyond T-ALL, a hierarchical axis is evident in multiple myeloma (MM), where Notch1 signaling transcriptionally upregulates MALAT1, which in turn functions to suppress the tumor-suppressive microRNA miR-125b, demonstrating a conserved lncRNA-dependent strategy for oncogene-mediated silencing of tumor suppressors (Gao et al., 2018). Collectively, these findings position the lncRNA-Notch interactome as a central, multi-layered command hub in hematologic malignancies, governing transcriptional programs, mediating adaptive pathway crosstalk, and enforcing oncogenic dependencies.

Hematologic malignancies provide the clearest example that ceRNA abundance in the literature does not necessarily indicate mechanistic primacy. In T-ALL and multiple myeloma, lncRNA-Notch crosstalk is dominated by promoter binding, enhancer looping, cis-regulation, feedback amplification, and adaptive pathway switching. These mechanisms place lncRNAs close to transcriptional control and oncogenic dependency, making this group particularly informative for studying non-ceRNA regulation.

### 4.6 Nervous system tumors

The regulatory patterns of nervous system tumors primarily rely on the synergistic effects of direct transcriptional regulation and ceRNA mechanisms. The interplay between lncRNAs and the Notch signaling pathway constitutes a sophisticated and clinically impactful regulatory network that drives the pathogenesis and therapeutic recalcitrance of nervous system tumors. This axis orchestrates a spectrum of malignant processes, from maintaining therapy-resistant cancer stem cells and promoting aggressive tumor progression to directly mediating chemotherapy resistance, with clear implications for patient prognosis.

In gliomas, the axis is fundamental for sustaining the glioma stem cell (GSC) population, a key determinant of treatment failure and recurrence. The lncRNA TUG1 operates as a critical effector, where its expression is induced by Notch1, creating a feed-forward loop that maintains GSC self-renewal through a dual mechanism of sponging miR-145 and recruiting epigenetic repressor (Katsushima et al., 2016). A more direct transcriptional control is exerted by FOXD2-AS1, which scaffolds the coactivator TAF1 to the Notch1 promoter, thereby enhancing its expression and reinforcing the stem cell state (Wang Y. et al., 2022). Beyond stemness, lncRNAs broadly activate the Notch pathway to fuel tumor progression. PlncRNA-1 upregulates multiple pathway components (Notch1, Jagged1, Hes1), while ZFAS1 promotes malignancy by modulating Notch signaling and EMT, with its high expression correlating with poor surviva (Gao et al., 2017; Wang X. et al., 2018). The regulatory complexity is heightened by intricate networks, such as the LINC01152/MAML2 positive feedback loop, where the lncRNA enhances the Notch co-activator MAML2, which in turn drives LINC01152 transcription, establishing a potent, self-reinforcing oncogenic circuit (Wu J. et al., 2021). The ceRNA mechanism is a dominant strategy, employed by LINC01410 and PVT1 to sponge miR-506-3p and miR-190a-5p/488-3p, respectively, leading to the derepression of Notch2 and the Notch ligand JAG1 (Xue et al., 2018; Zhao et al., 2022). This network also extends to immune modulation, as HOXA-AS2 upregulates JAG1 to foster an immunosuppressive microenvironment by expanding regulatory T-cells (Zhong et al., 2022). Conversely, the axis includes regulatory safeguards, evidenced by the tumor-suppressive lncRNA RAMP2-AS1, which inhibits Notch3 through an indirect mechanism (Liu et al., 2016).

Clinically, a paramount challenge is therapy resistance, a process in which the lncRNA-Notch axis is deeply implicated. In glioblastoma, LINC00021 is upregulated in temozolomide-resistant cells and executes a dual pro-survival strategy by activating Notch signaling while recruiting EZH2 to epigenetically silence the tumor suppressor p21 (Zhang S. et al., 2020). Although the mechanism for FEZF1-AS1 requires further elucidation, its upregulation of Notch-1 is associated with enhanced invasion and poor prognosis (Luo et al., 2020). This paradigm of lncRNA-mediated Notch activation driving treatment resistance is conserved in pediatric nervous system tumors with direct clinical consequences. In retinoblastoma, high expression of PVT1 and lincRNA-ROR correlates with advanced disease and recurrence. These lncRNAs function as ceRNAs, sponging miR-488-3p and miR-32-5p to upregulate Notch2 and Notch1, respectively, thereby promoting tumor progression and metastasis (Gao et al., 2021; Wu X. Z. et al., 2019). Similarly, TUG1 contributes to retinoblastoma malignancy via the Notch pathway (Wang H. et al., 2022). Most compellingly, in neuroblastoma, the axis is a direct mediator of cisplatin resistance. LINC00460 drives resistance by sponging miR-149-5p to elevate the Notch ligand DLL1 and activate Notch signaling—an effect that can be pharmacologically reversed by the Notch inhibitor DAPT, underscoring the direct therapeutic relevance of this axis (Xu et al., 2024).

Nervous system tumors highlight a stemness-centered pattern in which ceRNA regulation operates together with transcriptional and epigenetic control. TUG1, FOXD2-AS1, LINC01152, and LINC00021 connect Notch signaling with glioma stem cell maintenance, immune suppression, and temozolomide resistance. Pediatric tumors further extend this pattern to retinoblastoma and neuroblastoma, where Notch ligand or receptor derepression contributes to progression and cisplatin resistance. The key unresolved question is whether disrupting lncRNA scaffolding or feedback loops can more effectively eliminate resistant cancer stem-like populations than blocking Notch signaling alone.

### 4.7 Other systems

The lncRNA-Notch axis is established as a pervasive and mechanistically diverse oncogenic network, critically influencing tumorigenesis, metastasis, and therapy resistance. In melanoma, this axis exhibits remarkable complexity and specificity. Oncogenic lncRNAs precisely regulate distinct Notch receptors through at least two distinct mechanisms. IncRNAs BANCR and ZFPM2-AS1 promote progression by sponging miR-204 and miR-650, respectively, to derepress the Notch2 and Notch1 receptors (Cai et al., 2017; Liu et al., 2021). Furthermore, lncRNA BASP1-AS1 acts as a molecular scaffold in the nucleus, recruiting the transcription factor YBX1 to the Notch3 gene promoter to directly activate its transcription (Li et al., 2021c). This reveals a sophisticated network within a single tumor type where multiple lncRNAs fine-tune different nodes of the Notch pathway.

In osteosarcoma, this signaling axis plays a critical role in driving malignant progression and chemoresistance. The long non-coding RNAs CRNDE and SNHG12 promote tumor cell proliferation, invasion, and metastasis by modulating Notch1 and Notch2 signaling, respectively. (Li et al., 2018; Zhou et al., 2018). From a translational perspective, prognostic models based on multiple lncRNAs—specifically those associated with Notch signaling—can predict osteosarcoma metastasis and patient survival with high accuracy, macroscopically linking lncRNA signatures, Notch pathway activation, and poor prognosis (Zhang et al., 2021b).

Interestingly, in hemangioma (a benign tumor), the axis also plays a significant role and unveils a novel functional dimension. The functional versatility of lncRNA MEG8 is manifested through its regulation of Notch signaling via several ceRNA axes. Notably, it impacts cell proliferation through mechanisms involving the miR-203/JAG1-Notch1 axis and the miR-497-5p/Notch2 axis (Hu et al., 2021). Importantly, disruption of the MEG8/miR-497-5p/Notch2 axis leads to the downregulation of key ferroptosis inhibitors (SLC7A11/GPX4), thereby inducing ferroptosis, which for the first time connects this axis to a novel form of regulated cell death, expanding its functional scope (Ma et al., 2021).

Pancreatic neuroendocrine tumors exhibit a higher level of pathway crosstalk. Bioinformatics analyses identified the co-upregulation of lncRNA XLOC_221242 and the Notch-related receptor DNER. DNER expression positively correlates with the Notch core factor RBPJ and Wnt pathway components, suggesting the existence of a complex Wnt/Notch integrated signaling network, indicating that this axis may cooperate with other core pathways to drive tumorigenesis (He R. et al., 2020).

In conclusion, melanoma and osteosarcoma mainly show oncogenic receptor-level activation, hemangioma links the axis to ferroptosis, and pancreatic neuroendocrine tumors reveal integration with Wnt signaling. These examples reinforce the view that ceRNA mechanisms are common reporting units, whereas pathway scaffolding, transcriptional activation, cell-death regulation, and inter-pathway crosstalk may define the biological consequence in specific tumors.

## 5 Clinical translational perspectives and challenges

The lncRNA-Notch axis constitutes a multi-layered, multi-functional oncogenic network spanning benign to malignant diseases, involving single pathways to network interactions, and regulating basic phenotypes to cell death mechanisms. It represents a pool of highly promising diagnostic markers and therapeutic targets. However, its clinical translation as a non-invasive biomarker is still limited by technical bottlenecks.

In liquid biopsies such as plasma or extracellular vesicle lncRNA detection, there is currently a lack of standardized protocols for sample pre-processing, nucleic acid enrichment, and quantitative normalization. This leads to high heterogeneity in test results across different laboratories and makes batch effects highly likely, severely compromising reproducibility. Moreover, most candidate biomarkers have not yet undergone rigorous validation for absolute sensitivity and specificity in large-scale, multi-center prospective clinical cohorts (Ma et al., 2024). Without systematic performance evaluation tailored to the fluid-based testing environment, the actual clinical predictive value of these biomarkers is often overestimated.

At the therapeutic intervention level, targeted strategies against this regulatory axis have demonstrated remarkable therapeutic value in preclinical models. For instance, intervention with long non-coding RNA SOX2OT using natural compounds can effectively disrupt the downstream Notch3/DLL3 signaling cascade, thereby successfully reversing the stemness and chemoresistance of osteosarcoma in both *in vitro* and *in vivo* models (Wang W. et al., 2018).

However, the transition from preclinical proof-of-concept to practical clinical application must directly confront the failure lessons of traditional pathways and the delivery bottleneck unique to nucleic acid drugs. Historically, pan-Notch inhibitors represented by γ-secretase inhibitors (GSIs) severely disrupted normal intestinal epithelial homeostasis and induced irreversible gastrointestinal toxicity, forcing the termination of clinical trials of GSIs for most solid tumors (BeLow and Osipo, 2020; Sun et al., 2024). This lesson highlights the extremely high clinical risk of systemic Notch pathway blockade, and conversely confirms the rationality of targeting upstream-specific lncRNA to achieve “precision fine-tuning” of signaling.

Furthermore, a core challenge in translational medicine lies in addressing the delivery bottleneck that hinders the clinical advancement of RNA-targeted therapeutics. Although oncogenic lncRNAs have been established as promising molecular targets, the delivery of therapeutic RNA agents such as small interfering RNAs (siRNAs) and antisense oligonucleotides (ASOs) to tumor cells with high efficiency, specificity, and safety remains a critical barrier to clinical translation. Delivery platforms based on biocompatible nanocarriers, including exosomes and lipid nanoparticles (LNPs), are considered to possess high potential owing to their favorable biocompatibility, low immunogenicity, and emerging tissue-selective targeting capability (Wang X. et al., 2022). However, overcoming the delivery bottleneck in lncRNA-targeted therapy remains a core challenge. Current obstacles include large-scale production, stability, and targeted accumulation *in vivo*, necessitating optimization of pharmacokinetic properties through iterative carrier design and ligand development guided by computational biology. As highlighted in recent comprehensive reviews, mitigating carrier-induced immune activation, controlling regulatory safety margins for bidirectional networks, and minimizing sequence-dependent off-target effects are equally paramount to bridging the bench-to-bedside gap (Verma and Arora, 2025). More importantly, there is a significant validation gap in this field. Most studies remain confined to single models, lacking systematic validation across different cancer types and heterogeneous tumor microenvironments. Individual cases such as SOX2OT are insufficient to support generalizable conclusions. The lack of consistency between *in vitro* and *in vivo* functional effects within lncRNA regulatory networks represent an underappreciated critical shortcoming in current translational research. Bridging both technical and validation gaps is essential for translating preclinical findings into reliable clinical applications.

While the critical involvement of the lncRNA-Notch axis in malignancies is well-established, several limitations within the current literature merit attention. First, a distinct publication bias persists in this field, where negative or contradictory experimental results are frequently left unpublished in favor of positive oncogenic phenotypes. Second, substantial methodological heterogeneity—ranging from diverse cell line contexts to varying efficiencies of knockdown or overexpression strategies and functional assay parameters—hinders direct quantitative comparisons across independent studies. Lastly, much of the available clinical evidence remains heavily reliant on correlative clinical data rather than causal mechanistic validation in patient cohorts.

To overcome these constraints, future research should increasingly leverage rigorous systematic reviews and meta-analyses. Standardizing evaluation criteria across multi-center datasets can mathematically assess publication bias through funnel plot asymmetry tests. Furthermore, executing meta-regressions and integrating emerging single-cell multi-omics cohorts will better control for technical heterogeneity, effectively bridging the gap between correlative observations and true clinical causality.

## 6 Conclusion and prospect

The intricate bidirectional crosstalk between lncRNAs and the Notch signaling pathway constitutes a core regulatory axis of growing prominence in cancer biology. The significance of this axis lies not only in the role of lncRNAs as key upstream regulators of Notch pathway activity but also in their function as downstream effector molecules of the pathway’s transcriptional output, thereby forming complex feedback and feedforward loops that precisely control gene expression programs. This inherent property of interaction provides a solid foundation for their potential as novel prognostic biomarkers and highly promising RNA-targeted therapeutic strategies (Reicher et al., 2018).

Despite the wealth of evidence accumulated from preclinical studies, the translation of research findings on the lncRNA-Notch regulatory network into effective clinical strategies still faces multiple challenges, which chart a clear course for future investigations.

Primarily, future research must trace further upstream, aiming to elucidate the transcriptional initiation and epigenetic regulatory mechanisms of this regulatory network. Current studies on the lncRNA-Notch interaction predominantly focus on the mutual regulation between the two, while understanding of the core upstream events driving this network—namely, the transcriptional activation mechanisms of the oncogenic lncRNAs themselves—remains insufficient. For instance, in HCC, research has demonstrated that super-enhancers can drive the transcription of lncRNA HSAL3, subsequently activating Notch signaling (Yuan et al., 2023). This suggests that across different cancer types, there may be a prevalence of key lncRNAs regulated by specific epigenetic states, which act as critical upstream regulators of the Notch pathway. Utilizing cutting-edge technologies like chromatin conformation capture and CUT&Tag to systematically screen and identify these core lncRNAs, which are precisely regulated by epigenetics on a genome-wide scale, will help unravel the activation mechanism of this regulatory network at the level of transcriptional initiation and provide a theoretical basis for developing epigenetic drugs targeting the “network initiation hub.”

A tumor is a complex ecosystem comprising different cell subpopulations and a dynamically changing microenvironment. Traditional bulk sequencing techniques mask the specific functions of the lncRNA-Notch interplay across different cell types. Integrating technologies such as single-cell RNA sequencing, spatial transcriptomics, and CRISPR screening enables the precise mapping of the activity landscape of this regulatory network at single-cell resolution and identifies its specific roles in regulating processes like immune evasion, angiogenesis, or stromal remodeling within particular cellular subpopulations (Tien et al., 2020). This high-resolution analysis will uncover, previously unrecognized functions of the network within the tumor ecosystem—thereby establishing a mechanistic foundation for next-generation therapies that target key components of the TME, rather than cancer cells alone.

In conclusion, by deeply integrating innovative technologies, overcoming translational bottlenecks, and expanding the dimensions and depth of research, the systematic dissection of the lncRNA-Notch interactome will undoubtedly continue to inject new momentum into the precision diagnosis and treatment of cancer.

## Glossary

lncRNA: long non-coding RNA
ceRNA: competing endogenous RNA
miRNA: microRNA
mRNA: messenger RNA
Notch: neurogenic locus notch homolog protein
NICD: Notch intracellular domain
JAG1: Jagged canonical Notch ligand 1
JAG2: Jagged canonical Notch ligand two
DLL: Delta-like canonical Notch ligand
RBPJ: recombination signal binding protein for immunoglobulin kappa J region
Hes1: hairy and enhancer of split-1
EMT: epithelial-mesenchymal transition
OSCC: oral squamous cell carcinoma
LSCC: laryngeal squamous cell carcinoma
HCC: hepatocellular carcinoma
GBC: gallbladder cancer
CRC: colorectal cancer
NSCLC: non-small cell lung cancer
LUAD: lung adenocarcinoma
CC: cervical cancer
BC: breast cancer
TNBC: triple-negative breast cancer
OC: ovarian cancer
EC: endometrial cancer
PCa: prostate cancer
RCC: renal cell carcinoma
T-ALL: T-cell acute lymphoblastic leukemia
MM: multiple myeloma
GSC: glioma stem cell
TME: tumor microenvironment
MSC: mesenchymal stem cell
EV: extracellular vesicle
GSI: gamma-secretase inhibitor
siRNA: small interfering RNA
ASO: antisense oligonucleotide
LNP: lipid nanoparticle
CUT&Tag: cleavage under targets and tagmentation
